# Atelier paludisme: an international malaria training course held in Madagascar

**DOI:** 10.1186/1475-2875-7-80

**Published:** 2008-05-09

**Authors:** Olivier Domarle, Milijaona Randrianarivelojosia, Jean-Bernard Duchemin, Vincent Robert, Frédéric Ariey

**Affiliations:** 1 Institut Pasteur de Madagascar, BP 1274, (101) Antananarivo, Madagascar; 2 CERMES, BP 10887, Niamey, Niger; 3 IRD, UR-16 Vecteurs, and MNHN, USM-504 Biologie fonctionnelle des Protozoaires, Paris, France; 4 Institut Pasteur du Cambodge, Phnom Penh, Cambodge

## Abstract

The *Atelier Paludisme *(Malaria Workshop) is an international training course organized by the Institut Pasteur de Madagascar, which has been held annually for the past five years. The course was designed for both young and experienced researchers, as well as for healthcare professionals, mostly from malaria-endemic countries. Its objective is to provide participants with a broad knowledge of all features of malaria, to improve their skills in project management, to break geographical isolation by using the Internet as a source of documentary information. This six-week course makes use of concepts of andragogy and problem-based learning, i.e. a relationship between participants and tutors, which promotes a process of exchange rather than the simple transmission of knowledge, where participants have to search actively for information. This approach to training, combined with the wide background and experience of those involved, creates positive dynamics and enables participants to acquire new skills, develop their critical and analytical abilities. This paper describes the course and the lessons learned from its evaluation.

## Background

Malaria control programmes are based on several strategies, which work best when used in synergy. These strategies continually evolve as the result of technological advances, discovery of new tools, new drugs or new insecticides, as well as the results of innovative interventions carried out by experts. At country level, the efficacy of malaria control programmes depends on the establishment of coherent strategies, adapted to the specific needs of the country and with optimal use of limited resources.

For any strategy to be effective, the individuals involved need to have the best possible understanding not only of their own area of activity, but also of all the other technical disciplines involved in control programmes (e.g. diagnosis, treatment, epidemiology, entomology, education, communication, etc). In reality, at country-level, one finds that the various specialities involved in malaria control are often compartmentalized, and, even among decision-makers, knowledge of malaria is often limited and frequently out-of-date. The different actors, including clinicians, researchers and public health administrators, need to broaden their outlook beyond their own specialities in order to comprehend the problem as a whole. There are, currently, few courses that include the *'ability to comprehend the broader picture' *as their main training objective.

The *Atelier Paludisme*, developed at the Institut Pasteur de Madagascar, makes use of original training methods, including problem-based learning techniques and encouraging participants to play an active role in their own training, under the supervision of experts.

### Organization of the workshop

The general working principle of the *Atelier Paludisme *is to provide the participants with broadband internet access and enable them to find out about various aspects of malaria under the close supervision of international experts. The first week is devoted to practical sessions on improving computing skills and oral communication. Each of the following four weeks is organized around a major theme, and at the beginning of the week each participant is given a topic to research and expected, by the end of the week, to deliver a 10-minute presentation with power-point slides. The topics are selected in advance by the tutors, based on the topics covered each week and randomly distributed to the participants. Table 1 shows the overall course structure, including a selection of topics dealt-with by participants; full workshop content and participant presentations for the past five years can be found on the course web site [1]. The final week of the workshop is devoted to various forms of group work, including round table sessions and role-playing exercises. Practical sessions are organized at various times in the course of the workshop to illustrate technical issues (malaria diagnostic, geographical information systems, entomology).

**Table 1:** Breakdown of the programme

**Domains and situations**	**Number of hours**
Practical sessions	60 h
Presentations by tutors	30 h
Presentations by participants	
• Group work	36 h
• Individual work	82 h
• Presentations	32 h
• Assessment and individual evaluation	16 h

**TOTAL**	**256 h**

Details concerning the timetable for this course are published on the course website [1].

### Tutors and participants

In addition to course organizers, the teaching faculty consists of a group of 10–15 invited experts, who act as tutors to the participants. Each tutor stays with the course for one week (two or three tutors per week). The role of tutors is to guide the participants in their search for information on the internet, to help them to critically evaluate the information found and to make sure that their presentation is accurate (in order to avoid exposing the other participants to presentations containing erroneous information or misunderstandings).

Each tutor is asked to give a 50-minute lecture on a topic of their choice, usually their own research, and the lectures are followed by an open discussion with participants, course faculty and guests. These lectures by tutors represent the only formal teaching of the course and they represent only 10% of the overall contact time of about 256 hours.

**Figure 1:**
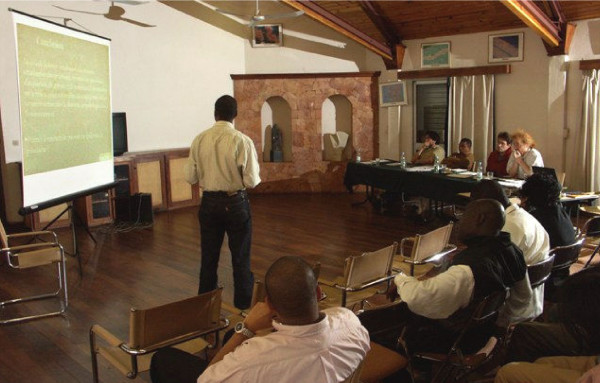
**Presentations to the panel**. Footnote: The presentations are given at the end of the week, in front of the other participants and the panel, composed of tutors. These sessions allow the participants to demonstrate their acquisitions, by defending their approach, and aim to evaluate the work done during the week (Photo: courtesy of Dr Olivier Domarle, *Atelier Paludisme *2006).

**Figure 2:**
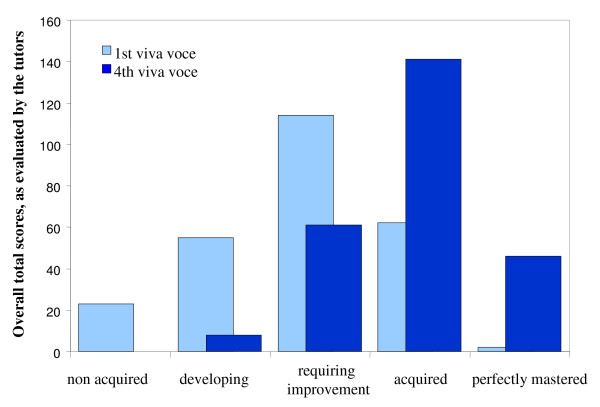
**Comparison of evaluations for the first and fourth presentations**. Footnote: Cumulative scores for all evaluation criteria are shown on the y axis. *Atelier Paludisme *2003; 13 participants.

At the end of each week, when participants give their 10-minute presentation, tutors and course organizers act together as a panel of examiners to evaluate the work and the presentation in a deliberately formal manner (Figure 1), using an evaluation grid including 20 criteria related to the form and content of the presentation, scored on a five-point scale (from "not acquired" to "completely mastered"). The scores awarded are used to construct a histogram providing a visual indication of the overall quality of the presentation and making it possible to identify strengths and weaknesses and to assess the progress made (Figure 2). The weekly cycle ends with an individual interview between each participant and the tutors for that week to identifying what the participant has learned and his/her weaknesses.

**Figure 3:**
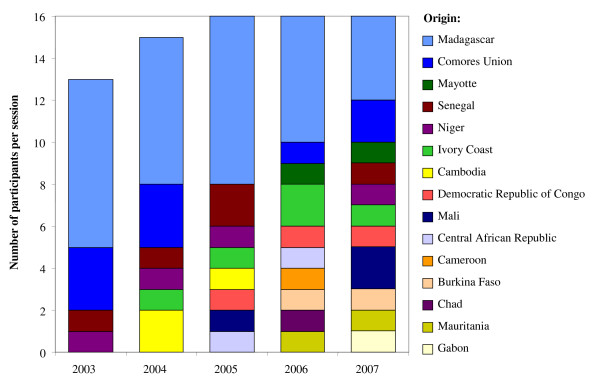
**Origin of the participants for the *Atelier Paludisme *sessions held in 2003–2007**. Footnote: The dissemination of information has led to an increase every year in the number and diversity of applicants. This increase has led to greater selection of applicants.

During the annual sessions from 2003 to 2007, 76 participants from 15 different countries were trained at the *Atelier Paludisme*. From the beginning, the intention was to achieve a good mix of participants: 1/3 clinicians, 1/3 public health administrators (i.e. scientists or health professionals involved in malaria control at national or regional level, usually employed by their Ministry of Health) and 1/3 research scientists (either at PhD student or post-doctoral level). Over the five-year period, this optimal ratio was more or less achieved. As the course was held in Madagascar, it is not surprising that 60% of its participants were from Madagascar for the first course in 2003, but this was reduced to 25% by 2007. As the course became increasingly well-known, the number of applicants gradually increased and so did the geographical diversity of participants with 11 different nationalities in 2007 (Figure 3).

### Evaluation and follow-up

Both tutors and participants were asked to evaluate the training, and their comments and suggestions were used to improve the workshop from one year to the next. An external audit of the five first sessions of the workshop was carried out in 2007, based on theoretical educational concepts. The design of this training was considered appropriate to its aims, particularly with respect to the acquisition of transverse skills, the use of appropriate learning models, the phases of knowledge acquisition, the identification of problems to be solved, material aspects, the availability of the tutors during the workshop, exchanges of experience between participants and tutors and the self-confidence acquired, especially when giving presentations. The preparation and delivery of presentations, and online bibliographical searches were considered important skills that the participants will be able to use in their professional lives, whatever their future post. The audit identified the following weak points, including a tendency to information overload, due to intensive weekly cycles preventing adequate consolidation of the knowledge acquired. This external viewpoint will be taken into account to improve future workshops.

In order to evaluate the long-term impact of the workshop, a follow-up of former participants was performed through annual questionnaires, to find out what impact the training had on their professional activities. In the 2007 survey, targeting participants having attended the first four sessions of the workshop, 68% (41/60) of participants responded. An overview of the responses of former participants and matched controls (who had attended different training courses in other subjects) is provided on Table 2. The subjective criteria recorded by former participants (improvements in their ability to assume their functions, strengthening of professional relationships) are positive, although one cannot exclude a bias in these responses.

**Table 2:** Follow-up of former participants and comparison with matched controls.

	Former participants	Matched colleagues
Responses	41/60 (68%)	19
Speciality:		
Doctor	46.3% (19/41)	53.6% (10/19)
Entomologist	12.2% (5/41)	15.8% (3/19)
Participant researcher	14.6% (5/41)	15.8% (3/19)
Pharmacist	7.3% (3/41)	
Specialist technician	4.9% (2/41)	5.3% (1/19)
Lecturer-researcher	4.9% (2/41)	
Other	9.8% (4/41)	10.5% (2/19)
Better able to assume functions since the training	Yes for 88% (36/41)	-
Better able to give presentations	Yes for 73% (30/41)	Yes for 58% (11/19)
Better able to train others	Yes for 80% (33/41)	Yes for 68% (13/19)
Better professional relations since the training	Yes for 85% (35/41)	-
Development of new activities since the training	Yes for 80% (33/41)	-
Development of a project as:		
Principal investigator	Yes for 34% (14/41)	Yes for 37% (7/19)
Collaborator	Yes for 68% (28/41)	Yes for 68% (13/19)
Obtained funding as:		
Principal investigator	Yes for 20% (8/41)	Yes for 21% (4/19)
Collaborator	Yes for 44% (18/41)	Yes for 37% (7/19)
Training has helped with grant applications and obtaining funding	Yes for 63% (26/41)	-
Change in professional activities since the training (as assessed by third party/superior)	Yes for 78% (32/41)	-

The survey of matched controls included colleagues of former participants. The questionnaires were distributed, by the former participants, to colleagues with similar training, in a similar post, dealing with malaria, tuberculosis or AIDS.

## Funding

The annual budget was approximately €80,000, covering the full costs for 15 to 16 participants and 10 to 15 invited tutors. It was considered essential to cover the cost for participants in order to be able to select them not on financial criteria, but on pertinent criteria such as past experience, country of origin, present involvement in malaria research or control. The Réseau International des Instituts Pasteur (the Institut Pasteur International Network) and Impact Malaria/Sanofi-Aventis have covered a large portion of this budget, with contributions from the Institut Pasteur de Madagascar, the Institut de Recherche pour le Développement, a French Overseas Development grant, the Direction des Affaires Sanitaires et Sociales de Mayotte, the World Bank, the WHO/Roll Back Malaria programme, the Agence Universitaire de la Francophonie and private partners (Fondation Mérieux, Natixis, Medical International and Technikon). This mixed funding reflects the variety of stakeholders needed for such a course and is important for its long-term viability.

## Conclusion

The *Atelier Paludisme *is currently one of the few courses to give a broad working knowledge of malaria and, as such, it meets the training needs of healthcare personnel involved in malaria research and control, enabling them to remain up-to-date with recent developments in the field. Because it focuses less on content and more on the resolution of problems and information finding, it contributes to honing the professional skills of participants. The *Atelier Paludisme *has recently been accepted as a module in the new *mastère spécialisé de l'École Pasteur/CNAM de santé publique *[2] and, since 2007, a sister-course in English is now run in Tanzania, as a collaboration between Institut Pasteur, the Swiss Tropical Institute and the Tanzanian Centre for International Training in Ifakara [3]. An expanded version of this paper was recently published in French [4].

## Authors' contributions

OD, MR, JBD, VR and FA were involved in initiating the *Atelier Paludisme*. All authors read and approved the final manuscript.
